# Discrepancies in Written Versus Calculated Durations in Opioid Prescriptions: Pre-Post Study

**DOI:** 10.2196/16199

**Published:** 2020-03-31

**Authors:** Benjamin H Slovis, John Kairys, Bracken Babula, Melanie Girondo, Cara Martino, Lindsey M Roke, Jeffrey Riggio

**Affiliations:** 1 Office of the Chief Medical Information Officer Thomas Jefferson University Philadelphia, PA United States; 2 Department of Emergency Medicine Thomas Jefferson University Philadelphia, PA United States; 3 Department of Surgery Thomas Jefferson University Philadelphia, PA United States; 4 Department of Medicine Thomas Jefferson University Philadelphia, PA United States; 5 Information Services and Technology Thomas Jefferson University Philadelphia, PA United States

**Keywords:** informatics, electronic health record, opioids, prescription, duration

## Abstract

**Background:**

The United States is in the midst of an opioid epidemic. Long-term use of opioid medications is associated with an increased risk of dependence. The US Centers for Disease Control and Prevention makes specific recommendations regarding opioid prescribing, including that prescription quantities should not exceed the intended duration of treatment.

**Objective:**

The purpose of this study was to determine if opioid prescription quantities written at our institution exceed intended duration of treatment and whether enhancements to our electronic health record system improved any discrepancies.

**Methods:**

We examined the opioid prescriptions written at our institution for a 22-month period. We examined the duration of treatment documented in the prescription itself and calculated a duration based on the quantity of tablets and doses per day. We determined whether requiring documentation of the prescription duration affected these outcomes.

**Results:**

We reviewed 72,314 opioid prescriptions, of which 16.96% had a calculated duration that was greater than what was documented in the prescription. Making the duration a required field significantly reduced this discrepancy (17.95% vs 16.21%, *P*<.001) but did not eliminate it.

**Conclusions:**

Health information technology vendors should develop tools that, by default, accurately represent prescription durations and/or modify doses and quantities dispensed based on provider-entered durations. This would potentially reduce unintended prolonged opioid use and reduce the potential for long-term dependence.

## Introduction

The United States is in the midst of an opioid epidemic [1,2]. The rate of death from opioids is increasing; more than 67% of the 70,237 overdose deaths in the United States in 2017 were attributed to opioid drugs [3]. Drug overdoses could soon become the number one cause of death of Americans under the age of 50 years [4]. Opioid overdoses are a public health crisis associated with significant medical costs and resource utilization [5-11]. Chronic opioid use is often preceded by treatment of acute pain [12], and long-term prescription opioids are associated with progression to illicit opioid abuse [13]. Despite the clear correlation between prescription opioid use and mortality, opioids continue to be routinely prescribed [2,14,15]. While the United States makes up only 4.3% of the global population [16], Americans consume more than 80% of the world’s supply of opioid medications [17].

In the Commonwealth of Pennsylvania, there were 5456 drug-related overdoses in 2017 [18], the third-highest of any state for drug-related deaths in the nation [19]. In 2016, 60% of all Pennsylvania counties had prescribing rates higher than the national average. In 2017, even though there was an overall decline in opioid prescriptions, enough oxycodone and hydrocodone were dispensed to provide every Pennsylvanian with 32 dosage units of these drugs [18].

Reduction in the number of opioid prescriptions, opioid doses, and duration of treatment are critical to curbing the opioid epidemic. State prescription drug monitoring programs (PDMPs) allow physicians to assess a patient’s controlled-substance prescription history [20]. Chronic opioid use and risk of death have been associated with higher prescription doses and longer durations [12,21]. After the third day of use in an opioid-naïve individual, the likelihood of chronic opioid use increases daily. This increase is most drastic on days 5 and 31 [22]. Therefore, the US Centers for Disease Control and Prevention (CDC) has authored guidelines on opioid dosing and duration for acute and chronic pain [23,24]. Specifically, they recommend a duration of 3 days or less for acute pain, and advise that more than 7 days is unlikely to be required. The CDC also recommends that prescriptions should be of no greater quantity than what is required for the duration of treatment [23]. Consequently, opioid prescriptions with unintentionally prolonged durations of treatment may result in increased risk of chronic opioid use and associated morbidity and mortality [25].

Health information technology can play a critical role in improving quality of care while improving guideline adherence and decreasing errors [26]. Electronic health records (EHRs) have been associated with advanced quality of patient care [27-29], and computerized provider order entry (CPOE) is associated with improved efficiency and safety [30]. CPOE can improve medication ordering through clinical decision support systems in both active (ie, a pop-up warning of drug-drug interactions) and passive (ie, a dose-appropriate default prescription setting) approaches [31].

Some health information technology interventions that require providers to review aspects of current or prior opioid prescriptions have been demonstrated to affect opioid prescribing patterns. For instance, PDMP mandates that require the review of controlled substance prescription history prior to and during opioid prescribing have demonstrated a reduction in opioid prescribing rates [32]. Additionally, while some hospitals have attempted to control opioid prescribing patterns through prescription presets of a specific number of tablets [33,34], others have demonstrated both reductions and increases in tablets dispensed when requiring manual entry of number of tablets to dispense in a prescription [35,36].

Our current vendor-based EHR’s CPOE ordering screen has four fields for opioid prescription entry: (1) dose (ie, number of tablets), (2) frequency (eg, three times a day), (3) duration (ie, number of doses or days), and (4) quantity (ie, the number of tablets dispensed). Previously, the system did not auto-calculate duration from dose, frequency, and quantity or vice versa. Instead, it alerted the provider within the CPOE ordering screen and recommended updating the duration or quantity. This means a provider could write a prescription with a duration of 3 days and a quantity of 200 tablets with only a soft alert. Additionally, prior to August 2018, our institution did not require a duration value to be entered into our CPOE, meaning this field could be left blank. In August 2018, we introduced a number of interventions via modifications to our EHR’s opioid prescription settings: (1) duration was set to be a required field, (2) a *quick button* for 3 days’ duration was added to coincide with CDC guidelines for acute pain, and (3) tablet quantity for all opioid orders was preset to 10.

The purpose of this research is to examine if there are differences between the duration of treatment as written in an opioid prescription versus the duration associated with the dosing frequency and number of pills dispensed. That is to say, we examined how long the number of dispensed pills at the prescribed frequency would last (ie, the *calculated duration*) and compared that to the duration documented by the prescriber (ie, the *written duration*) for opioids ordered by providers at our institution. Additionally, we examined whether the requirement of a value in the duration field—from the interventions described above—had any effect on accurately representing the *calculated duration* of the prescription. We take special interest in those durations that are calculated to be longer than what was written in the duration field, as these directly contradict the CDC prescribing guidelines [23] and may increase risk of the associated negative effects of prolonged opioid use.

## Methods

### Data Acquisition

We queried the EHR system of the Center City division of our health care system, which includes an urban academic tertiary care center, an urban academic community hospital, and multiple ambulatory clinics. We examined data generated by Epic (Epic Systems Corporation) via a third-party analytics software, Qlik Sense (QlikTech International), to develop a list of all outpatient opioid prescriptions, including discharge medications, written over 22 months from October 2017 to July 2019. This includes an 11-month preintervention period and an 11-month postintervention period.

We extracted a number of variables, including the quantity of tablets, the dosage units (ie, mg), the route of administration (ie, oral vs buccal), the *discrete dose* (ie, 15 mg vs 10-15 mg, based on the number of tablets per dose), the written duration, and the frequency of administration.

For the purposes of this study, we limited the route of administration to *oral* and excluded all nontablet formulations. Finally, we excluded the medications buprenorphine and methadone, as these are routinely used for the management of opioid use disorder.

### Calculated Duration

In order to compute the calculated duration of each prescription, we took each of the unique possible frequencies in the system (ie, twice a day or every 4 hours) and mapped these to the equivalent number of administrations per 24-hour day. For pro re nata (PRN) medications, we selected the maximum frequency possible. Therefore, “twice a day PRN” resulted in two daily administrations and “every other day” resulted in a daily administration of 0.5.

Next, we took all doses that contained ranges for their *discrete dose* (ie, 20-30 mg) and isolated the maximum dose possible per administration (ie, 30 mg). We calculated the number of tablets per administration by dividing the maximum dose by the dose per tablet. We calculated the total number of possible doses by dividing the quantity dispensed by number of tablets per administration. Finally, we computed the calculated duration, measured in days, by dividing the total number of doses by the number of administrations per 24 hours. The choice of the maximum dose per administration means that our calculated duration is the shortest possible when holding the other variables constant.

### Statistical Analysis

We first converted all written durations to be represented in units of *days*. We then examined the proportion of prescriptions that did not have a written duration to determine if our intervention had an effect on the documentation of this field. We also examined the proportion of prescriptions for which we could not compute a calculated duration to ensure the preintervention and postintervention periods were similar. Finally, we compared the number of prescriptions written with ranges (ie, 20-30 mg) to see if our intervention had an effect on this category of prescriptions.

To compare what was documented (ie, written duration) to what was dispensed (ie, calculated duration) for each prescription, we then generated our study cohort by excluding any prescription that did not hold a value for both of these fields. Also, since we computed the calculated duration in units of days, we excluded all written durations not also documented in units of days.

We examined the written duration of each prescription and compared it to the calculated duration. We categorized each relationship as a written duration equal to, greater than, or less than the calculated duration. We examined whether there were any changes in the relationships between written duration and calculated duration before and after our interventions, with specific interest regarding the requirement to document a written duration. See Table 1 for examples of each field and how we coded relationships.

**Table 1:** Examples of each field extracted from the electronic health record system, with written duration and calculated duration demonstrated, along with their relationship.

Drug	Prescription instructions	Dispense quantity	Written duration	Administrations per day	Maximum dose per administration	Calculated duration^a^	Relationship
Morphine: 15 mg tablets	Take 1-2 tablets as needed twice a day for 3 days	12	3 days	2	30 mg (2 tablets)	3 days	Written duration is equal to calculated duration
Morphine: 15 mg tablets	Take 1-2 tablets as needed twice a day for 3 days	8	3 days	2	30 mg (2 tablets)	2 days	Written duration is longer than calculated duration
Morphine: 15 mg tablets	Take 1-2 tablets as needed twice a day for 3 days	20	3 days	2	30 mg (2 tablets)	5 days	Written duration is shorter than calculated duration

^a^Calculated duration=((quantity of tablets × drug dose in mg) / maximum dose per administration) / administrations per day.

Data cleaning and calculations were performed in Qlik Sense. Statistical analysis was performed in the statistical software R, version 3.3.2 (The R Foundation). Chi-square analysis was used for categorical values. The *t* test was used for parametric data and the Wilcoxon rank-sum test was used for nonparametric data.

## Results

### General Results

There were 92,462 unique opioid prescriptions that met initial inclusion criteria during our study period for 30,426 individual patients. There was a mean number of prescriptions per month of 4202.82 (SD 204.32) and a median number of prescriptions per person per month of 1 (IQR 1-2).

In the preintervention period, there were 47,131 prescriptions for 16,863 individual patients; in the postintervention period, there were 45,331 prescriptions for 17,483 individual patients. There was no significant difference (*P*=.06) between mean number of prescriptions per month in the preintervention period (mean 4284.64 [SD 200.68]) versus the postintervention period (mean 4121.00 [SD 180.74]). The median number of 1 prescription per person per month did not change (IQR 1-2, *P*=.37).

There was a statistically significant decrease in the proportion of prescriptions with no written duration documented postintervention (33.54%, 95% CI 33.12-33.97, vs 9.45%, 95% CI 9.19-9.72, *P*<.001). Evaluation of the remaining 9.45% of prescriptions without a written duration appear to be due to refills of prior prescriptions, which were exempt from the new documentation requirement.

There was a small but significant difference in the proportion of prescriptions in which we were unable to compute the calculated duration when comparing the preperiod and the postperiod (6.01%, 95% CI 5.80-6.23, vs 3.61%, 95% CI 3.44-3.79, *P*<.001).

There was no significant difference pre- and postintervention in the number of prescriptions that contained ranged doses (ie, 20-30 mg) (4.42%, 95% CI 4.23-4.61, vs 4.66%, 95% CI 4.47-4.86, *P*=.08).

**Figure 1:**
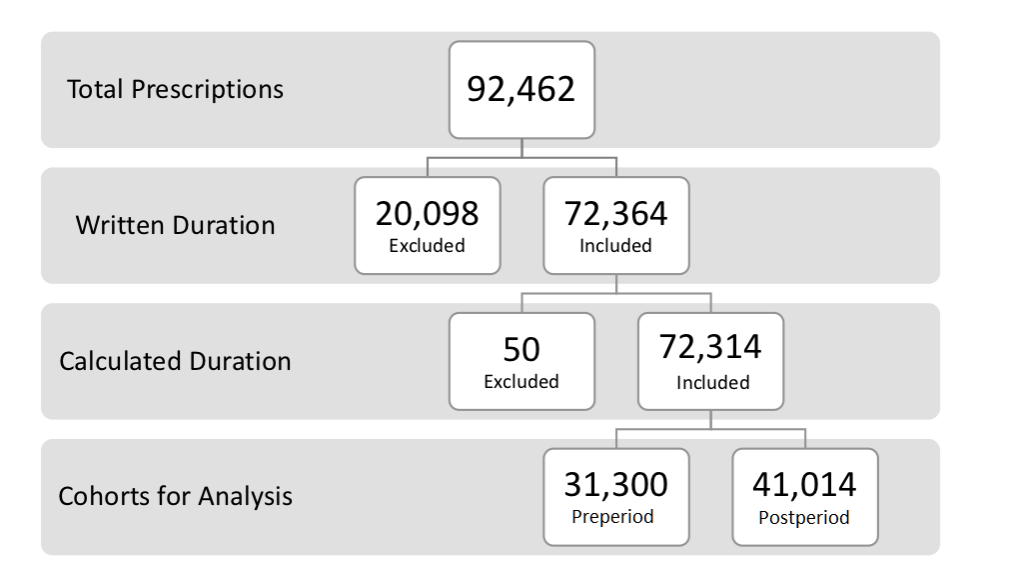
Breakdown of cohorts based on inclusion and exclusion criteria and separation into preperiod and postperiod cohorts.

There were 72,364 out of 92,462 (78.26%) total prescriptions that had a written duration documented. Of these, there were 2632 (3.64%) prescriptions whose written duration units were converted from other duration units to *days*, while the rest were already written in this unit of measurement. Out of 72,364 prescriptions, 50 (0.07%) were excluded because the calculated duration could not be computed. This resulted in 72,314 out of 92,462 (78.21%) total prescriptions meeting the inclusion criteria for our comparison: out of 72,314 prescriptions, 31,300 (43.28%) were in the preintervention period and 41,014 (56.72%) were in the postintervention period. Figure 1 demonstrates the inclusion and exclusion criteria and separate cohorts for comparison.

### Comparison of Written and Calculated Durations

Of the 72,314 prescriptions, 41.97% (30,353 total, 95% CI 41.61-42.33) had calculated durations that were equivalent to their written durations, 41.06% (29,694 total, 95% CI 40.70-41.42) had calculated durations that were less than their written durations, and 16.96% (12,267 total, 95% CI 16.69-17.24) had a calculated duration that was greater than the written duration.

Requiring a written duration to be documented significantly improved the number of calculated durations that were equal to their corresponding written duration, from 38.86% (12,163/31,300, 38.86%, 95% CI 38.32-39.40) to 44.35% (18,190/41,014, 44.35%, 95% CI 43.87-44.83, *P*<.001). Additionally, requiring a written duration resulted in a reduction in prescriptions where the calculated duration was longer than the written duration (5617/31,300, 17.95%, 95% CI 17.52-18.37, vs 6650/41,014, 16.21%, 95% CI 15.86-16.57, *P*<.001). Changes in percentages of each relationship between written duration and calculated duration, pre- and postintervention, are presented in Table 2.

**Table 2:** Totals and percentages of each cohort and their associated relationships for pre- and postintervention.

Relationship	Preperiod (N=31,300), n (%)	Postperiod (N=41,014), n (%)	*P* value	Total (N=72,314), n (%)
Calculated duration equal to written duration	12,163 (38.86)	18,190 (44.35)	<.001	30,353 (41.97)
Calculated duration less than written duration	13,520 (43.19)	16,174 (39.44)	<.001	29,694 (41.06)
Calculated duration greater than written duration	5617 (17.95)	6650 (16.21)	<.001	12,267 (16.96)

## Discussion

### Principal Findings

The opioid epidemic is a major concern in the United States, specifically in the Commonwealth of Pennsylvania [1,18] where the governor has issued a state emergency disaster declaration since 2018 [37]. Our hospitals and clinics are located in the heart of a major urban area within the state, in a county that hosts the highest estimated frequency of overdose deaths [38] and neighbors a district considered to be the epicenter for illicit opioid trade in the region [39].

In order to aid in many state-, city-, and hospital-level initiatives, our clinical informatics group has recently employed solutions to combat the opioid epidemic. These include simplifying PDMP queries, modifications of prescription settings in the emergency department, and analytics tools to track opioid prescribing throughout the institution [40,41].

Reduction in opioid prescriptions is a key step in combating this epidemic, and the duration of prescriptions has a significant effect on a patient’s risk of becoming a chronic opioid user [12,22].

In order to study our upcoming planned interventions, we realized we needed a reliable way to calculate durations of opioid prescriptions. Given the prior lack of requirement to document a written duration in our EHR system, we wanted to explore whether this was an accurate representation of a prescription’s true duration and whether making this a requirement improved overall representation of the duration of a prescription as measured by the calculated duration.

Overall, written duration was *equal to* the calculated duration (41.97%) or *less than* the calculated duration (41.06%) the majority of the time. However, 16.96% of the time our prescriptions had a longer calculated duration than the written duration, which contradicts CDC prescribing guidelines. We are concerned that unintentionally prolonged opioid prescription durations could be contributing to an increased risk of opioid dependence. We must take this opportunity to stress that we do not imply that these prescriptions were intentionally written to break guidelines or to prolong a patient’s duration of opioid therapy. We are simply stating that we observed a difference in these two values and we believe this requires attention. One solution to this issue could be modifications to the EHR system.

In order to provide peak levels of patient safety and end-user satisfaction, EHRs require continuous review and optimization. A lack of a required field allowed prescription durations to not match what the provider had documented, or likely intended. This is apparent in that requiring documentation of this field reduced those prescriptions without written durations, improved the percentage of written durations that accurately represented their corresponding calculated durations, and reduced the number of calculated durations that exceeded written durations. As discussed in our results, we explored the remaining 9.45% of prescriptions that continued to lack a written duration after our intervention and determined that they were likely due to renewals of prescriptions written prior to our modifications, where the system allows the field previously left blank to remain. Additionally, our intervention did yield a small but significant reduction in prescriptions where we were unable to compute a calculated duration (6.01% vs 3.61%). We attribute this to the addition of a quick button, as described in our methods.

Many studies have demonstrated how modifications to CPOE can affect opioid prescribing, though most of the literature focuses on tablet counts and not duration of treatment. Delgado et al demonstrated that when transitioning from an EHR that had no preset dispense quantity to an EHR that required a preset of 10 tablets, the median number of oxycodone 5 mg/ acetaminophen 325 mg tablets dispensed by two emergency departments decreased from 11.3 to 10.0 and from 12.6 to 10.9, respectively [33]. Similarly, Chiu et al demonstrated that when opioids were prescribed at discharge for outpatient surgery, modification of the default pill count from 30 tablets to 12 tablets reduced the median number of tablets dispensed from 30 to 20 tablets [34]. However, they did not examine prescription duration, noting that duration guidelines are usually “far longer than most patients will need,” and modifications of number of tablets dispensed will have a more profound affect [34]. While we believe reduction of tablets dispensed is critical to combating the opioid epidemic, at this time, CDC guidelines consider prescription duration to be an important measurement in reducing opioid therapy and combating the epidemic [23,24].

Conversely, other studies have demonstrated results from entirely removing presets for opioid tablets dispensed. Santistevan et al showed that in an emergency department setting, removal of the default of 20 tablets for hydrocodone and oxycodone, and the requirement of the prescriber to enter a number of tablets to dispense, reduced the median number of tablets prescribed from 20 to 15 [35]. The authors concluded that EHR presets may hinder providers’ ability to prescribe opioids to appropriately fit the variability in patient care (ie, more painful clinical conditions need more tablets) and that prescriptions written at the provider’s discretion may be more appropriate. Contrary to these results, Zwank et al demonstrated an increase in mean number of tablets from 15.31 to 15.77 after removing their 15-tablet preset and requiring manual entry of tablets dispensed [36].

Crothers et al also examined the effects of transitioning between EHR systems, from a homegrown system to a vendor-based system [42]. Their prior EHR system auto-calculated the maximum number of *dispense units* for PRN opioid prescriptions based on dose, frequency, and duration documented. During their implementation, they removed this functionality and instead developed preset dispense quantities, with outpatient prescriptions for clinics and inpatient discharges defaulted to 30 tablets for oxycodone and *null* for hydrocodone. This resulted in a decrease of 1.4 dispense units overall and 3.9 dispense units for inpatient discharge [42].

While these studies have examined the effect of changes in the requirement to document the number of tablets dispensed [33-36], to our knowledge we are the first to examine the requirement of documenting a duration field and comparing the duration intended by a prescriber (ie, written duration) to the calculated duration based on the number of tablets dispensed and instructions provided.

Our results confirm that simple documentation of an opioid prescription’s duration is not sufficient. When a duration field is not directly linked to other elements of the prescription and there is no automated calculation of duration from dose, frequency, and quantity—or vice versa—inaccuracies remain. While it is interesting that Crothers et al demonstrated that removal of such functionality reduced opioid dispensing [42], this was performed during a transition of EHR systems and with the addition of prescription presets, which likely influenced their results. Given the importance of the duration of an opioid prescription in the associated risk of long-term use, as well as further potential for overdose, it is vital to have data that accurately represents this value. Additionally, we have demonstrated that retrospective computation of a calculated duration is a viable alternative when written durations are not available for the study of EHR prescribing data.

In order to improve compliance to CDC opioid prescribing guidelines, EHR system vendors should consider rapidly developing tools that, by default, accurately represent prescription durations and/or modify doses and quantities dispensed based on provider-entered durations. As described in the Introduction, this calculation already exists in our system, but no hard stop or passive updating of prescription fields exists. If a provider intends to prescribe one tablet, three times a day for 3 days, the system should automatically set the quantity at nine tablets; if the provider reduces the quantity to six tablets, the duration of the prescription should automatically be reduced to 2 days.

### Limitations

Our study was performed at a single, urban academic institution and its associated ambulatory clinics; therefore, our results may not represent the majority of the country’s hospitals. However, given that our institution is located in the state with the third-highest rate of drug overdose, our results may be valid when compared to other states of similar rates of overdose.

Additionally, as discussed in our methods, we intentionally biased our process of computing the calculated duration to make our resulting durations as short as possible by assuming individual doses were the higher of the possible range (ie, if a possible dose was 5-10 mg, we assumed the dose was 10 mg, thereby meaning that the quantity was used faster and the duration was shorter). This means that our calculations may underrepresent the number of prescriptions where the calculated duration was longer than the written duration if patients were to ration their medications and take lower doses over a longer period of time. Further, while our main intervention was making the duration field a requirement, we also added quick-action buttons and modified some prescription settings, which may have influenced our postperiod results, though we expect this to have been minimal. Finally, we used EHR data to represent durations of medications. While we believe it is important for our prescriptions to accurately represent the intended therapy and to abide by CDC guidelines, we did not determine whether individual patients took their prescribed medication as written, nor did we examine whether each prescription was filled at a pharmacy.

### Conclusions

Accurate documentation of an opioid prescription’s duration is critical, both for patient safety and for secondary use in analysis of the status of the opioid epidemic, as well as for evaluating interventions implemented to combat this public health crisis. Our study demonstrates that more than 17% of prescriptions written at our institution had durations documented in the EHRs that were shorter than durations calculated via the dose, frequency, and quantity of tablets prescribed. Requiring documentation of the duration field in a prescription improved these errors statistically but, clinically, a large number of prescriptions continued to not match the calculated duration. EHR vendors should invest in research and development to create functions that automatically calculate and fill values of the opioid prescription to ensure prescriptions are accurately represented, while physicians and hospitals should invest in informatics initiatives to study and improve provider-prescribing practices.

## Abbreviations

CDC: US Centers for Disease Control and Prevention
CPOE: computerized provider order entry
EHR: electronic health record
PDMP: prescription drug monitoring program
PRN: pro re nata
